# Subcutaneous Trastuzumab: An Observational Study of Safety and Tolerability in Patients With Early HER2-Positive Breast Cancer

**DOI:** 10.1155/2024/9551710

**Published:** 2024-06-22

**Authors:** Iris Otoya, Natalia Valdiviezo, Zaida Morante, Cindy Calle, Yomali Ferreyra, Norma Huarcaya-Chombo, Gabriela Polo-Mendoza, Carlos Castañeda, Tatiana Vidaurre, Silvia P. Neciosup, Mónica J. Calderón, Henry L. Gomez

**Affiliations:** ^1^ Department of Medical Oncology Instituto Nacional de Enfermedades Neoplásicas, Lima, Peru; ^2^ Department of Bioengineering Universidad de Ingeniería y Tecnología, Lima, Peru; ^3^ Faculty of Science and Engineering Universidad Peruana Cayetano Heredia, Lima, Peru; ^4^ Institute of Investigations in Biomedical Sciences (INICIB) Universidad Ricardo Palma, Lima, Peru; ^5^ Oncosalud Auna Ideas, Lima, Peru

## Abstract

**Purpose:** In Peru, breast cancer (BC) stands as the most predominant malignancy neoplasm among women. Trastuzumab has marked a significant milestone in the management of this disease. It has been shown to improve prognosis in human epidermal growth factor receptor 2 (HER2)–expressing female patients, but its repercussions and efficacy are yet to be analyzed in a context with limited resources.

**Methods:** The study population is made of woman patients aged 18 years and older diagnosed with HER2-positive BC at Instituto Nacional de Enfermedades Neoplásicas (INEN, Lima, Peru) during 2019–2021 and treated with at least one dose of subcutaneous trastuzumab. We reviewed medical records to register treatment characteristics, adverse events (AEs), disease progression, and survival status. We considered a median follow-up time of 36 and 45 months for progression and survival status.

**Results:** The majority of patients were over 50 years old (54.29%). Tumor size averaged 19.7 ± 16.1 mm. Lymph nodes were present in 44.78% of patients. Most patients received adjuvant chemotherapy (63.8%) as first-line treatment. Descriptive analyses of treatment outcomes revealed a 30% toxicity rate, primarily attributed to arthralgia (47.62%), followed by diarrhea, fatigue, and injection site reactions, with relatively lower discontinuation rates compared to larger scale studies. Differences in demographic, clinical, and treatment characteristics were not statistically significant concerning the emergence of AEs (*p* > 0.05). Progression appeared in nine patients, and the overall survival (OS) rate stood at 98.6% and 92.8%, respectively, during a median follow-up of 36 and 45 months.

**Conclusion:** The research suggests that subcutaneous trastuzumab is comparable in effectiveness and safety to the intravenous administration. Regional-specific studies may provide valuable insights into demographic factors influencing treatment outcomes in Peru or other countries. Furthermore, it could represent a more accessible alternative, potentially enhancing patient adherence and optimizing healthcare resource logistics.

## 1. Introduction

Breast cancer (BC) has become the most frequently diagnosed malignant neoplasm among Peruvian women [1]. It is particularly predominant in Metropolitan Lima, with an incidence of 18.2% and a mortality rate of 12.7 per 100,000 women, causing 2135 deaths during the 2013–2015 period [2]. These statistics underscore the relevance and the need to effectively address the diagnosis and treatment of BC in the context of public health in Peru.

BC is characterized by its heterogeneous nature, presenting diverse histological types and target structures. Among the subtypes identified, human epidermal growth factor receptor 2 (HER2)–positive BC and triple-negative BC stand out as the most aggressive histologies with unfavorable prognoses [3]. The former owes its aggressivity to the overexpression of HER2, which plays a significant role in tumor development and progression [4].

Trastuzumab is used in HER2-targeted therapy and acts by inhibiting the HER2 signaling pathway that drives cell growth [5]. It was first approved in 1998 for treating metastatic BC and in 2006 for HER2-positive early BC by the US Food and Drug Administration (FDA), marking a significant breakthrough in BC management [6]. Combining intravenous trastuzumab with standard chemotherapy has been shown to extend the time to progression and improve survival in HER2-expressing female patients [7, 8]. However, given the routine continuation of this treatment for 1 year in cases of early HER2-positive BC, efforts were made to reduce the time and optimize delivery. This endeavor led to the development of a subcutaneous formulation in 2013, based on the results of the HannaH study in neoadjuvant and adjuvant treatment of early HER2-positive BC [8].

This subcutaneous route could be a more convenient and easier-to-use alternative. The benefit of subcutaneous administration has been examined, and several studies have shown that, in patients previously treated with intravenous trastuzumab, subcutaneous presentation is preferred due to shorter treatment times, reduced use of healthcare resources, and greater convenience for patients and, consequently, improved patient quality of life [9–11]. Likewise, subcutaneous trastuzumab is not inferior in performance to its counterpart, according to the primary endpoints of pathologic complete response, such as the absence of invasive neoplastic cells in the breast area and ductal carcinoma remaining in situ [12]. Furthermore, their efficacy is not altered by administration; follow-up analyses at 3 years demonstrated similar event-free survival (EFS) and overall survival (OS) between both presentations [13]. Although the safety of the treatment has been previously confirmed [10], the safety and tolerability profile of subcutaneous trastuzumab for patients with HER2-positive BC while under real-world conditions in a context of limited human resources, medicines, and infrastructure, such as Peru [14], remains to be determined.

This study intends to assess the safety and tolerability of subcutaneous trastuzumab administered in a neo/adjuvant setting in a Peruvian population of patients with HER2-positive BC employing a clear and reproducible methodology. Our results will provide comprehensive insight into the most commonly reported adverse effects and reasons for treatment discontinuation. Additionally, we describe patients' responses to treatment in terms of progression. Given Peru's limited resources and current healthcare system, ensuring equitable, safe, and effective BC treatment is crucial. This research optimizes resource utilization, enhances patient safety, contributes valuable data to global knowledge, and informs healthcare policies tailored to the unique challenges of resource-limited settings.

## 2. Methods

### 2.1. Study Population

The study focuses on patients at the Instituto Nacional de Enfermedades Neoplásicas (INEN, Lima, Peru) receiving oncological care. The participants are female, of mixed race, aged 18 or older, covered by public health insurance, and diagnosed with clinical stage I to IIIC positive HER2 BC, with an Eastern Cooperative Oncology Group (ECOG) performance status of 0–1.

These patients were diagnosed at INEN between 2019 and 2021 and received at least one dose of subcutaneous trastuzumab in a neo/adjuvant setting. The prescribed regimen was a fixed dose of 600 mg every 3 weeks for 18 cycles.

The exclusion criteria were as follows:

• Patients partially treated at another institution
• Patients carrying a second malignancy
• Patients without confirmation of HER2 positivity

Exclusion criteria resulted in a population of 70 patients. All patients in the study population were sampled, and the sampling was nonprobabilistic by convenience.

### 2.2. Variables and Data Collection

Medical records were reviewed to collect demographic data such as age, civil status, and region of precedence. Human development index (HDI) per region by the Instituto Peruano de Economía from 2019 was used as a way to evaluate differences in socioeconomic status and educational level; values under 0.550, between 0.555 and 0.699, and between 0.700 and 0.799 were classified as low, medium, and high HDI [15].

We also recorded clinical characteristics such as tumor size (mm), lymph node status, TNM classification, neo/adjuvant first-line treatment, treatment completion status, previous use of intravenous trastuzumab, and adverse events (AEs). We analyzed and scored the emergence of AEs during the treatment period up to 28 days after the last treatment according to the National Cancer Institute's Common Terminology Criteria for Adverse Events (NCI—CTCAE), version 5.0 [16].

We registered progression and survival status at a median follow-up time of 36 and 45 months for both. The National Registry of Identification and Civil Status (RENIEC) was consulted to determine the survival status of patients. Median follow-up time was considered from the date of diagnosis to the last medical appointment or date of death, as appropriate.

We created a database using Microsoft Excel 2000 (Microsoft Corporation, USA) to allow cross-referencing and export to statistical programs. The data will be available upon request.

### 2.3. Statistical Analysis

Continuous variables, such as age, tumor size, lymph nodes, treatment duration, and number of cycles received, were described by mean and standard deviation. Categorical variables, such as first-line treatment setting, treatment completion, presence of AEs, first AEs, survival status, progression status, and progression site, were described by number and percentage of patients in each category, using either Wilcoxon rank sum test, Fisher's exact test, or Pearson's chi-squared test as appropriate. All statistical analyses were performed using R software version 4.03, packages “gtsummary” and “tidyverse.” A statistical significance threshold of *p* ≤ 0.05 was established for all tests.

### 2.4. Ethical Aspects

This research project was approved by the INEN Project Review Committee. Patients' informed consent was not required as the retrospective approach involved no more than minimal risk to the subjects. The data obtained is exclusively for academic research purposes, and the confidentiality of the information obtained has been respected at all times, from data collection, storage, and processing to the dissemination of the study results. No information related to the identity of the participants has been made public.

### 2.5. Limitations and Feasibility

Despite our efforts to develop a rigorous methodology, limitations must be acknowledged. The retrospective nature of the study introduces biases in data collection, including the potential for incomplete or inaccurate medical records. Additionally, the lack of a control group restricts our ability to make direct comparisons and draw causal conclusions. Furthermore, the small sample size (70 patients) and the single-center nature of the study may limit how much our results can apply to a broader context or different healthcare settings. The small sample size was also an obstacle to subgroup analyses based on treatment setting (neoadjuvant vs. adjuvant) and could have influenced comparison by the presence of AEs. Using nonprobabilistic sampling may have also introduced selection bias, as patients were not randomly selected but chosen based on data availability.

Despite these limitations, the study contributes valuable insights. It can serve as a basis for future research within the context of the healthcare system in Peru and even in settings of similar conditions. Moreover, we employed real-world data from INEN, a national reference and research institution for cancer, highlighting the importance of local context and resource limitations in optimizing oncology care. Finally, future research could be built upon our findings by conducting large-scale studies with multiple institutions, more extended follow-up periods, and incorporating control groups to further elucidate the efficacy and safety profile of subcutaneous trastuzumab.

## 3. Results

### 3.1. Demographic Characteristics

Seventy female patients diagnosed with HER2-positive BC were treated with subcutaneous trastuzumab and incorporated into the analysis. All of them were mixed-race females covered by public health insurance (Sistema Integral de Salud or SIS).

**Figure 1:**
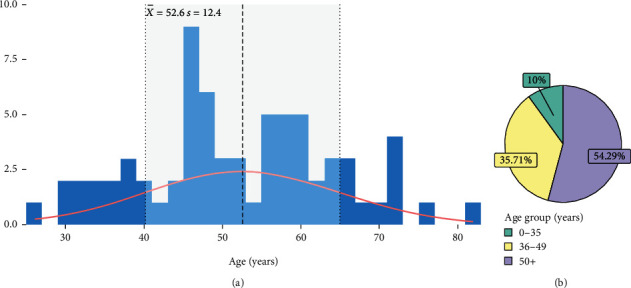
Distribution of diagnosed patients by (a) age and (b) age group.

The mean age population was 52.6 ± 12.4 years with a median of 51.5 years, but no specific distribution pattern could be discerned (Figure 1(a) and Table 1). Considering age groups, 54.29% (*n* = 38) of patients were over 50 years old, 35.71% (*n* = 29) were between 36 and 49 years, and 10.0% (*n* = 7) of women were 35 or under that age (Figure 1(b) and Table 1). In the study population, the HDI distribution varied across categories, with 71.4% falling into the high HDI group, 4.3% into the medium HDI group, and 24.3% into the low HDI group. Regarding civil status, most patients were single (72.9%). A smaller proportion was married (17.1%), divorced (7.1%), and widowed (2.9%) (Table 1).

### 3.2. Disease Characteristics

**Figure 2:**
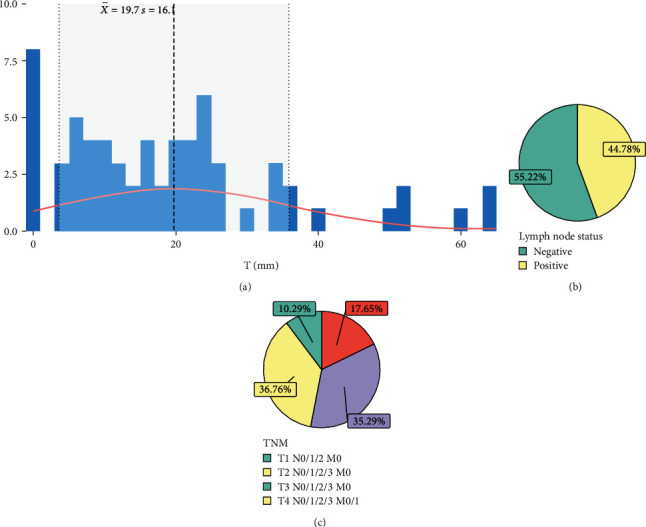
Distribution of clinical characteristics by (a) tumor size frequency, (b) lymph node status, and (c) TNM classification.

The median tumor size was 17.5 mm, with a range of 8.0–25.3 mm and a mean size of 19.7 ± 16.1 mm, although no specific distribution pattern could be discerned following this parameter (Figure 2(a) and Table 1).

Regarding lymph node status, 55.22% of patients had negative lymph nodes, while 44.78% had positive lymph nodes (Figure 2(b) and Table 1). In terms of TNM classification, 10.29% of patients were mainly reported as T2 N0/1/2/3 M0 (36.76%), followed by T3 N0/1/2/3 M0 (35.29%), T4 N0/1/2/3 M0/1 (17.65%), and T1 N0/1/2 M0 (10.29%) (Figure 2(c) and Table 1).

### 3.3. Treatment

**Figure 3:**
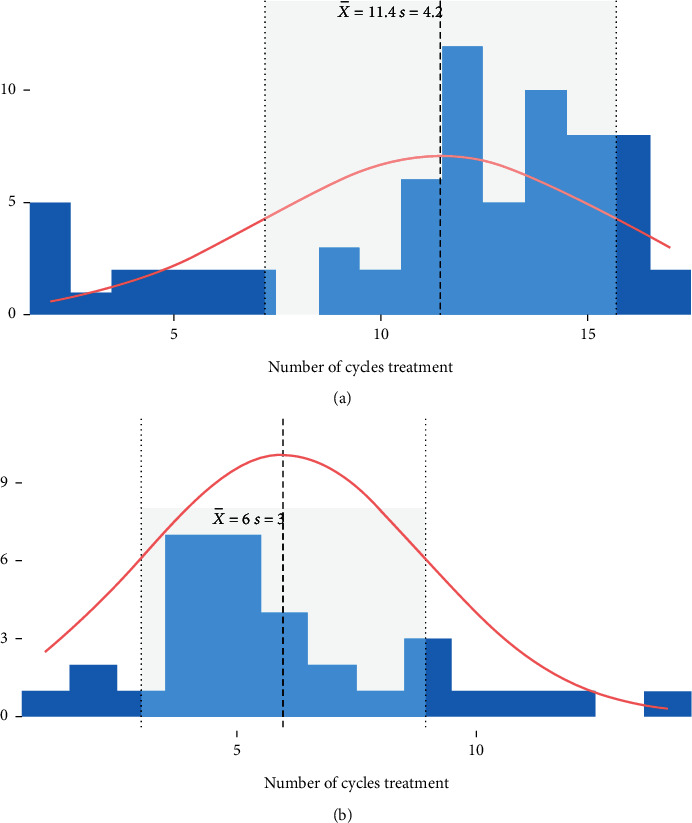
Distribution of patients by quantity of subcutaneous trastuzumab treatment cycles in (a) the general population and (b) the subpopulation that received intravenous trastuzumab prior to subcutaneous.

Of all patients, 63.8% (*n* = 44) started treatment with subcutaneous trastuzumab in the adjuvant setting, while the remaining 36.2% (*n* = 25) incorporated it in the neoadjuvant setting (Table 2). The mean number of cycles of trastuzumab received subcutaneously was 11.4 ± 4.2, but no pattern could be discerned in its distribution (Figure 3(a)).

Out of 68 patients (two missing cases), only 2.9% (*n* = 2) had to discontinue trastuzumab treatment by subcutaneous administration due to severe toxicity. Cardiotoxicity and hypertension were the reasons why patients chose to discontinue their treatment (Table 2). It must also be noted that some patients did not start trastuzumab treatment subcutaneously but intravenously (45.71%, *n* = 32, Table 2). These patients received an average of 6 ± 3 cycles (Figure 3(b)).

### 3.4. Safety

**Figure 4:**
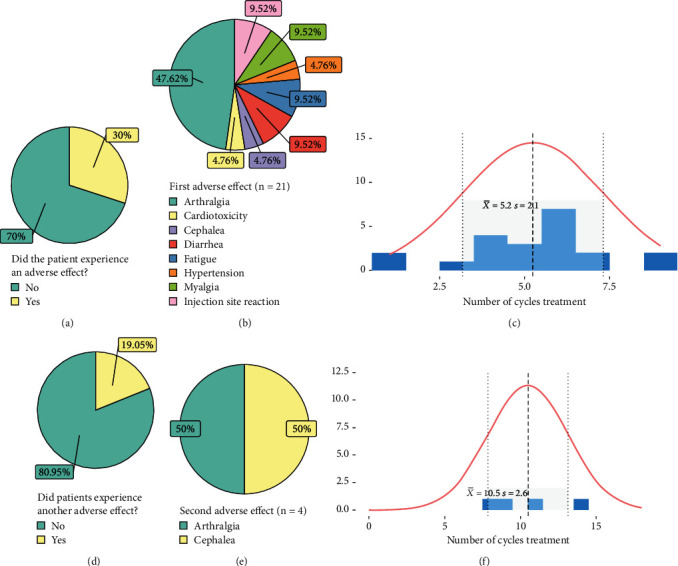
Emergence of adverse events with subcutaneous trastuzumab administration: (a) presence of first event; (b) type of first adverse event; (c) number of cycles received before the first event occurs; (d) presence of second event; (e) type of second adverse event; (f) number of cycles received before the second event occurs.

Thirty percent of patients (*n* = 21; Figure 4(a) and Table 2) experienced AEs such as arthralgia (*n* = 10, 47.62%), diarrhea (*n* = 2, 9.52%), fatigue (*n* = 2, 9.52%), myalgia (*n* = 2, 9.52%), and injection site reaction (*n* = 2, 9.52%). Less frequent AEs include cardiotoxicity, cephalea, and hypertension (Figure 4(b) and Table 2). The mean number of cycles received until the first occurrence of AE appeared was 5.2 ± 2.1 (Figure 4(c)). Of the patients who experienced the first AE, only 19.05% (*n* = 4) experienced side effects again (Figure 4(d)), where only cases of arthralgia and headache were reported (Figure 4(e)). Patients received around 10.5 ± 2.6 cycles of treatment until that moment (Figure 4(f)). Demographic and clinical characteristics were evaluated to assess their relation to the emergence of AEs, but no variable was statistically significant (Table S1). Likewise, treatment characteristics and patient status did not affect the presence of AEs, although the frequency of patients that did not report AEs was higher in those with prior intravenous treatment than the group that only received subcutaneous administration (53.1% vs. 46.9%); this was not significant (Table S2).

### 3.5. Survival Outcomes

**Figure 5:**
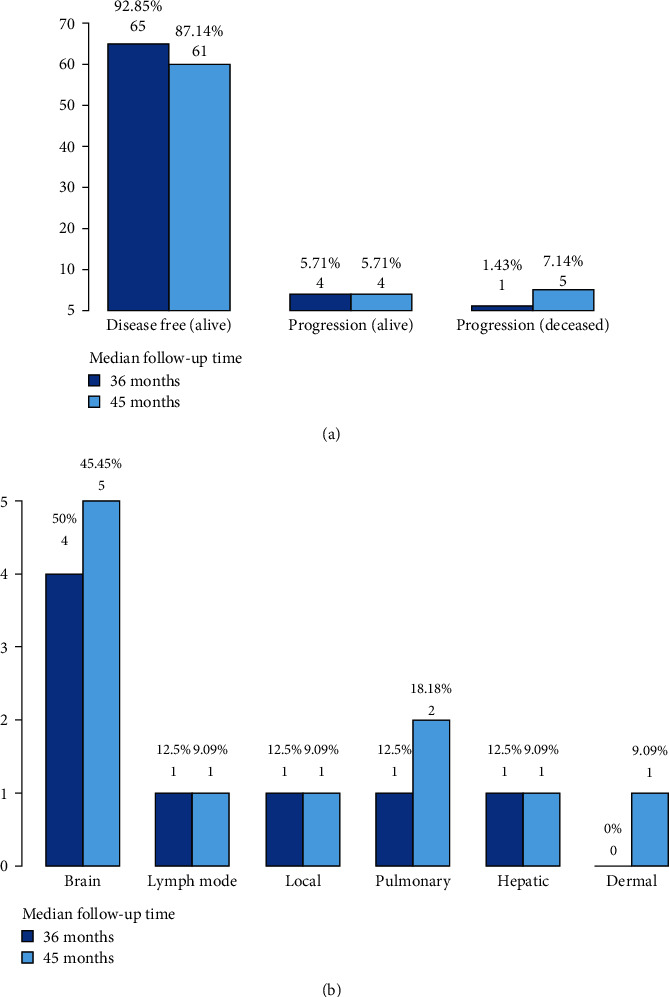
Treatment outcomes at 36 months and 45 months: (a) patient status and (b) type of progression.

Follow-up data was recovered from 69 patients. At a median follow-up time of 36 months, five patients had developed progression (7.1%), and one of them died (1.4%), giving a 98.6% of surviving patients. At 45 months, another four patients developed progressions (5.7%), and those with progression at 36 months died (5.8%), given a total of five deaths (7.2%). The remaining 64 patients (92.8%) were still alive at the time of analysis, but only 61 (87.1%) had a complete response (Figure 5(a) and Table 2). When evaluating if patient status was affected due to treatment completion or progression development, we only found an association with the latter (*p* < 0.001). All five patients who died had developed progression previously (Table 3). Progression occurred mainly at the brain level, alone or with another malignancy. At 36 months, the disease progressed to the brain, lymph nodes, local region, lungs, and liver. At 45 months, the disease also progressed to the dermal level (Figure 5(b)).

## 4. Discussion

Both intravenous and subcutaneous trastuzumab have shown potency in improving HER2-positive BC prognosis [7, 17]. Our findings suggest adequate safety and efficacy with subcutaneous trastuzumab, even in limited-resource settings.

The median age of our study population was 52.6 years, slightly lower than the median age reported in other studies [18–20], even though HER2 overexpression cancer is infrequent in older patients [21]. A study of German patients with advanced BC found a median age of 59.5 years [18]. In an Australian cohort of older women with HER2-positive metastatic BC, the median age was 73 years [19].

The different median ages may occur because of the most advanced BC stage since, in another study focused on patients with early HER2+ BC, the median age was 54 years [22]. However, it may also reflect variations in the study populations and underscores the importance of considering demographic factors in evaluating HER2-positive BC treatment. Our analysis showed no significance in the emergence of AEs regarding age groups (*p* = 0.074; Table S1). Moreover, while some studies suggest an adequate safety of trastuzumab in older patients [21, 22], others reported an increased risk of cardiotoxicity with age [20, 23].

These age-related differences in medication response may stem from changes in pharmacodynamic responses, which tend to occur as individuals age and experience physiological alterations [24]. Additionally, studies on subcutaneous trastuzumab have demonstrated pharmacokinetic similarities to intravenous trastuzumab, with comparable terminal half-lives (8 mg/kg vs. 6 mg/kg, respectively, and approximately 10 days for both) [25]. Moreover, bioavailability for both administrations is similar, roughly 70%–90% [25–27]. Therefore, subcutaneous trastuzumab appears to be an appropriate alternative route administration.

Since demographic characteristics such as educational level, economic status, and social support tend to be associated with better coping and treatment compliance [28, 29], we intended to use IDH and civil status to assess if they are associated with suspension and disease progression. However, only two patients in our population suspended treatment. This number is so small that comparisons were discouraged. Regardless, previous evidence has shown that old age, household responsibilities, and distance to travel are most important in determining noncompliance to treatment in rural settings [30]. This is important to note because while INEN is a national oncological reference center, its location in Lima could make traveling back and forth for treatment between the province and the capital difficult. These demographic data provide insight into the backgrounds of the study participants, which may play a role in their access to healthcare services, treatment outcomes, and overall well-being. Further research that considers potential biases and limitations is needed.

Studies such as SafeHER and others have demonstrated an acceptable safety profile of subcutaneous trastuzumab internationally, with a survival benefit of around 5 years [31, 32]. This aspect has yet to be extensively evaluated in Peru with a real population, underlining the need and importance of our research in this specific context. Our study expects to bring more clarity to this panorama.

We identified a mild to moderate toxicity rate of 30% with subcutaneous trastuzumab. This proportion differs greatly from that found in an Asian population, where 88.7% presented AEs of any grade and 23.3% severe [33]. Our patients predominantly presented arthralgia, diarrhea, fatigue, and reaction in the injection site, with 19.05% of them experiencing recurrent side effects. Although frequencies differ, similar side effects have been found previously in a study that enrolled patients from 59 countries (19.2%, 20.1%, and 19.9%, respectively) [34] and in Germany (8.0%, 8.6%, and 12.8%, respectively) [35]. Treatment with intravenous trastuzumab has shown similar AEs, with both administrations having less than 5% cardiotoxicity and AEs being grade 1 or 2 [12, 34]. Less common AEs were infections, cephalea, reaction at the injection site, gastrointestinal disorders, vascular disorders, and cardiac disorders [34, 36–38], which, excluding infections, our patients demonstrated.

However, in some studies, PrefHer and HannaH trials showed low and comparable rates of serious AEs between subcutaneous and intravenous treatments, but a comprehensive analysis of three randomized controlled trials revealed a higher incidence of AEs with subcutaneous trastuzumab, mainly related to the injection site [12, 34, 39]. These contrasts suggest remarkable variability in the tolerability of subcutaneous administration in different populations, highlighting the importance of region-specific studies. Our findings indicate a possible greater sensitivity to subcutaneous trastuzumab related to injection sites in our cohort, possibly due to differences in demographic characteristics or clinical management practices. It is important to note that in some studies, subcutaneous administration caused more AEs than the intravenous one, but these tended to be nonserious [8, 38, 40, 41], and in the case of severe AEs, their quantity was not statistically different [8]. In summary, our safety results are consistent with previous works [8, 34, 42, 43] and do not raise concerns by comparison with intravenous trastuzumab. In addition, severe AEs were more frequent in an adjuvant setting [35].

Moreover, for the management of these events, if grade 3 or 4, it is suggested to hold on treatment until reaching grade 1 and reducing dose [44], especially in cardiac events where symptomatic congestive heart failure needs a permanent discontinuation of trastuzumab [44]. In our finding, trastuzumab-induced arthralgia was the most frequent AE; it is suggested to treat with steroid-sparing csDMARD or bDMARD, prednisolone, or nonsteroid anti-inflammatory drug (NSAID)/non-NSAID analgesics, depending on toxicity grade [45]. Gastrointestinal problems can be treated with dexamethasone or delayed nausea prophylaxis. Regarding fatigue, other treatable causes should be discarded, and patients must be informed of what to expect from treatment [44].

SCHEARLY and SafeHER studies reported early discontinuation of only 7.5% and 5.1%, respectively, of subcutaneous trastuzumab [11, 32]. Likewise, our cohort showed that only 2.86% had to discontinue treatment due to severe AEs related to cardiac AEs, as in previous work [34]. A German study noted a discontinuation rate of 1.2%, also related to cardiac events, including ejection fraction decreased, cardiac failure, and atrial fibrillation [35]. Given the original small sample size of our study, this finding must be treated with caution.

Studies such as HannaH trial indicate that subcutaneous trastuzumab can achieve a similar ratio of pathologic complete response to intravenous administration (45.4% and 40.7%, respectively) [8] and in neoadjuvant setting (41.5%) [35], with comparable EFS and OS rates between both formulations [13, 46, 47]. A study that evaluated EFS and OS for up to 6 years showed 65% and 84% rates, respectively, for both groups [12]. These findings are lower than our results but similar enough given our smaller sample group and follow-up time (3 years). We have observed an OS and PFS rate of 87.1% and 92.8%, respectively, with a median follow-up of 45 months, results that align better with rates at a similar follow-up time of 94.9% OS and 74.8% PFS at 42 months [42], 90.5% OS at 4 years [38], and 90.8% EFS [35]. In another phase III HannaH clinical trial, subcutaneous trastuzumab competed adequately with the standard intravenous formulation, with comparable OS and EFS rates between groups [13]. This suggests that the high survival observed in our study could be related to the inherent efficacy of trastuzumab, regardless of its form of administration. Likewise, another study in a large cohort of Italian women treated with trastuzumab showed very favorable OS rates, further supporting the efficacy of trastuzumab in the clinical setting [48]. This contrast reflects not only the efficacy of trastuzumab in the treatment of HER2-positive BC but also the importance of good tolerability and a manageable safety profile, which are essential to ensure treatment adherence and thus improve survival outcomes in this patient population.

Most of our patients experienced brain progression. Previous studies have indicated that up to 50% of patients with HER2-positive BC may develop brain metastases during the course of the disease [49–51]. It is essential to consider the limited ability of trastuzumab to cross the blood-brain barrier, which restricts its effectiveness in controlling extracranial metastases [52]. Despite advances in anti-HER2 therapies, brain metastases in HER2-positive BC remain a significant cause of morbidity and mortality [53]. In this context, results from the TUXEDO-1 trial evaluating trastuzumab deruxtecan, an antibody-drug conjugate with significant extracranial activity, observed an intracranial response rate of 73.3% in patients with active brain metastases [54]. In addition, trastuzumab deruxtecan has shown efficacy in patients with HER2-positive metastatic BC who had progressed after prior therapies, including T-DM1 [55]. These findings suggest that antibody-drug conjugates may have relevant clinical activity in brain metastases, possibly due to an altered blood-brain barrier at the site of metastases.

According to our study, 45.7% of patients initially received intravenous trastuzumab, subsequently opting for the subcutaneous form. This finding is aligned with the existing literature, as demonstrated by the PrefHer study, which indicates that the transition between intravenous and subcutaneous forms of trastuzumab does not alter its known safety profile [32]. This aspect is relevant; as it suggests that the choice of administration method can be adapted to patients' preferences and needs without compromising the efficacy or safety of the treatment. It also highlights the importance of considering patient comfort, since patients tend to favor subcutaneous trastuzumab over intravenous trastuzumab, even switching treatments, due to more comfort and less time associated with treatment administration [56, 57]. Additionally, there is a greater preference of staff for subcutaneous trastuzumab due to potential cost and time savings [58], around 603,000 EUR and 1100 h in a Swedish study [59], which highlights its clinical relevance in situations of scarce resources. As such, a Peruvian study highlights the lack of comparative cost data but suggests economic advantages for subcutaneous trastuzumab, especially relevant in contexts of limited funding [60].

Our findings provide valuable insight into the administration of trastuzumab, highlighting the importance of personalizing treatment and considering demographic characteristics when evaluating the efficacy and safety of therapies. Based on this study, more extensive research could be initiated on the efficacy and quality of life in various treatment modalities. Comparative studies of clinical outcomes and quality of life between the two routes of administration in patients with HER2-positive cancer could be carried out, as well as cost-effectiveness analyses that consider both direct and indirect costs, including the impact on the patient's quality of life.

## 5. Conclusions

This study addresses therapeutic efficacy and resource optimization in oncology treatment. First, it reaffirms the safety of subcutaneous trastuzumab in a real-world setting, supporting data from controlled clinical trials. This external validation is crucial for the widespread acceptance of therapies. Secondly, it highlights the possibility of reducing the length of hospital stay, which would not only improve the patient's quality of life but would also optimize the use of health system resources, a relevant aspect considering the limited availability of these resources.

## Figures and Tables

**Table 1:** General characteristics of patients.

**Characteristic**	**N**	**Overall (** **N** = 70^a^**)**
Age (years)	70	51.5 (46.0, 60.8)
Age group (years)	70	
18–35		7 (10.0%)
36–49		25 (35.7%)
50+		38 (54.3%)
HDI	70	
High		50 (71.4%)
Medium		3 (4.3%)
Low		17 (24.3%)
Civil status	70	
Single		51 (72.9%)
Divorced		5 (7.1%)
Married		12 (17.1%)
Widowed		2 (2.9%)
Tumor size (mm)	64	17.5 (8.0, 25.3)
NR		6
Lymph node status	67	
Negative		37 (55.2%)
Positive		30 (44.8%)
NR		3
TNM classification	68	
T1 N0/1/2 M0		7 (10.3%)
T1 N0 M0		2
T1 N1 M0		4
T1 N2 M0		1
T2 N0/1/2/3 M0		25 (36.8%)
T2 N0 M0		8
T2 N1 M0		12
T2 N2 M0		2
T2 N3 M0		3
T3 N0/1/2/3 M0		24 (35.3%)
T3 N0 M0		2
T3 N1 M0		11
T3 N2 M0		10
T3 N3 M0		1
T4 N0/1/2/3 M0/1		12 (17.6%)
T4 N0 M0		2
T4 N1 M0		5
T4 N1 M1		1
T4 N2 M0		2
T4 N3 M0		2
NR		2

Abbreviation: NR, not reported.

^a^Median (IQR); *n* (%).

**Table 2:** Treatment status and tolerability.

**Characteristic**	**N**	**Overall (** **N** = 70^a^**)**
First-line treatment	69	
Adjuvant chemotherapy		44 (63.8%)
Neoadjuvant chemotherapy		25 (36.2%)
NR		1
Treatment status	68	
Completed		66 (97.1%)
Suspended		2 (2.9%)
Cardiotoxicity		1
Hypertension		1
NR		2
Intravenous trastuzumab treatment	70	
No		38 (54.3%)
Yes		32 (45.7%)
Adverse events	70	
No adverse events		49 (70.0%)
Reported adverse events		21 (30.0%)
Arthralgia		10
Cardiotoxicity		1
Cephalea		1
Diarrhea		2
Fatigue		2
Hypertension		1
Myalgia		2
Injection site reaction		2
Progression status	70	
Complete response at 45 months		61 (87.1%)
Progression		9 (12.9%)
At 36 months		5
At 45 months		4
Survival status	69	
Alive at 45 months		64 (92.8%)
Deceased		5 (7.2%)
At 36 months		1
At 45 months		4
NR		1

Abbreviation: NR, not reported.

^a^Median (IQR); *n* (%).

**Table 3:** Characteristics related to patient status at 45 months.

**Characteristic**	**N**	**Overall (** **N** = 69^a^**)**	**Alive (** **N** = 64^a^**)**	**Deceased (** **N** = 5^a^**)**	**p** **value** ^b^
Treatment status	67				> 0.9
Suspended		1 (1.5%)	1 (1.6%)	0 (0.0%)	
Completed		66 (98.5%)	63 (98.4%)	3 (100.0%)	
Unknown		2	0	2	
Progression status	69				< 0.001
No		60 (87.0%)	60 (93.8%)	0 (0.0%)	
Yes		9 (13.0%)	4 (6.3%)	5 (100.0%)	

^a^Median (IQR); *n* (%).

^b^Wilcoxon rank sum test; Fisher's exact test.

## Data Availability

The data used to support the findings of this study are available from the corresponding author upon request.

## Supporting Information

Supporting Information 1 Table S1 General characteristics by emergence of adverse events.

Supporting Information 2 Table S2 Treatment characteristics and patient status by emergence of adverse events.
