# Supplementary material for: Subcutaneous Trastuzumab: An Observational Study of Safety and Tolerability in Patients With Early HER2-Positive Breast Cancer
Source: Int J Breast Cancer. 2024 Jun 22;2024:9551710. doi: 10.1155/2024/9551710 (PMC11222001; doi:10.1155/2024/9551710)
Supplement: Supporting Information 1 — Table S1 General characteristics by emergence of adverse events. [file 9551710.f1.docx]

**Supplementary Table 1:** General characteristics by emergence of adverse events

| **Characteristic** | **N** | **Overall**, | **No AEs**, | **Reported AEs**, | **p-value**^2^ |
| --- | --- | --- | --- | --- | --- |
|  |  | N = 70^1^ | N = 49^1^ | N = 21^1^ |  |
| **Age (years)** | 70 | 51.5 (46.0, 60.8) | 52.0 (41.0, 61.0) | 49.0 (47.0, 60.0) | 0.6 |
| **Age group (years)** | 70 |  |  |  | 0.074 |
| 18-35 |  | 7 (10.0%) | 7 (14.3%) | 0 (0.0%) |  |
| 36-49 |  | 25 (35.7%) | 14 (28.6%) | 11 (52.4%) |  |
| 50+ |  | 38 (54.3%) | 28 (57.1%) | 10 (47.6%) |  |
| **HDI** | 70 |  |  |  | 0.6 |
| High |  | 50 (71.4%) | 33 (67.3%) | 17 (81.0%) |  |
| Low |  | 17 (24.3%) | 13 (26.5%) | 4 (19.0%) |  |
| Medium |  | 3 (4.3%) | 3 (6.1%) | 0 (0.0%) |  |
| **Civil status** | 70 |  |  |  | 0.13 |
| Divorced |  | 5 (7.1%) | 3 (6.1%) | 2 (9.5%) |  |
| Married |  | 12 (17.1%) | 8 (16.3%) | 4 (19.0%) |  |
| Single |  | 51 (72.9%) | 38 (77.6%) | 13 (61.9%) |  |
| Widowed |  | 2 (2.9%) | 0 (0.0%) | 2 (9.5%) |  |
| **Tumor size (mm)** | 64 | 17.5 (8.0, 25.3) | 19.0 (8.5, 26.5) | 17.0 (6.5, 23.5) | 0.3 |
| NR |  | 6 | 3 | 3 |  |
| **Lymph node status** | 67 |  |  |  | 0.6 |
| Negative |  | 37 (55.2%) | 27 (57.4%) | 10 (50.0%) |  |
| Positive |  | 30 (44.8%) | 20 (42.6%) | 10 (50.0%) |  |
| NR |  | 3 | 2 | 1 |  |
| **TNM classification** | 68 |  |  |  | 0.2 |
| T1 N0/1/2 M0 |  | 7 (10.3%) | 5 (10.4%) | 2 (10.0%) |  |
| T2 N0/1/2/3 M0 |  | 25 (36.8%) | 14 (29.2%) | 11 (55.0%) |  |
| T3 N0/1/2/3 M0 |  | 24 (35.3%) | 19 (39.6%) | 5 (25.0%) |  |
| T4 N0/1/2/3 M0/1 |  | 12 (17.6%) | 10 (20.8%) | 2 (10.0%) |  |
| NR |  | 2 | 1 | 1 |  |
| ^1^Median (IQR); n (%) | | | | | |
| ^2^Wilcoxon rank sum test; Fisher's exact test; Pearson's Chi-squared test | | | | | |
| Adverse events (AEs) | | | | | |
| Not reported (NR) | | | | | |
