# Supplementary material for: Subcutaneous Trastuzumab: An Observational Study of Safety and Tolerability in Patients With Early HER2-Positive Breast Cancer
Source: Int J Breast Cancer. 2024 Jun 22;2024:9551710. doi: 10.1155/2024/9551710 (PMC11222001; doi:10.1155/2024/9551710)
Supplement: Supporting Information 2 — Table S2 Treatment characteristics and patient status by emergence of adverse events. [file 9551710.f2.docx]

**Supplementary Table 2:** Treatment characteristics and patient status by emergence of adverse events

| **Characteristic** | **N** | **Overall**, | **No AEs**, | **Reported AEs**, | **p-value**^2^ |
| --- | --- | --- | --- | --- | --- |
|  |  | N = 70^1^ | N = 49^1^ | N = 21^1^ |  |
| **First-line treatment** | 69 |  |  |  | 0.8 |
| Adjuvant chemotherapy |  | 44 (63.8%) | 31 (64.6%) | 13 (61.9%) |  |
| Neoadjuvant chemotherapy |  | 25 (36.2%) | 17 (35.4%) | 8 (38.1%) |  |
| NR |  | 1 | 1 | 0 |  |
| **Treatment status** | 68 |  |  |  | 0.092 |
| Suspended |  | 2 (2.9%) | 0 (0.0%) | 2 (9.5%) |  |
| Completed |  | 66 (97.1%) | 47 (100.0%) | 19 (90.5%) |  |
| NR |  | 2 | 2 | 0 |  |
| **Intravenous Trastuzumab treatment** | 70 |  |  |  | 0.059 |
| No |  | 38 (54.3%) | 23 (46.9%) | 15 (71.4%) |  |
| Yes |  | 32 (45.7%) | 26 (53.1%) | 6 (28.6%) |  |
| **Progression status** | 70 |  |  |  | 0.4 |
| Progression at 36 months |  | 5 (7.1%) | 5 (10.2%) | 0 (0.0%) |  |
| Progression at 45 months |  | 4 (5.7%) | 3 (6.1%) | 1 (4.8%) |  |
| Complete response at 45 months |  | 61 (87.1%) | 41 (83.7%) | 20 (95.2%) |  |
| **Survival status** | 69 |  |  |  | >0.9 |
| Deceased at 36 months |  | 1 (1.4%) | 1 (2.0%) | 0 (0.0%) |  |
| Deceased at 45 months |  | 4 (5.8%) | 3 (6.1%) | 1 (5.0%) |  |
| Alive at 45 months |  | 64 (92.8%) | 45 (91.8%) | 19 (95.0%) |  |
| NR |  | 1 | 0 | 1 |  |
| ^1^Median (IQR); n (%) | | | | | |
| ^2^Wilcoxon rank sum test; Fisher's exact test; Pearson's Chi-squared test | | | | | |
| Adverse events (AEs) | | | | | |
| Not reported (NR) | | | | | |
